# Using Longitudinal Assessment on Extensively Managed Ewes to Quantify Welfare Compromise and Risks

**DOI:** 10.3390/ani8010008

**Published:** 2018-01-08

**Authors:** Carolina Munoz, Angus Campbell, Stuart Barber, Paul Hemsworth, Rebecca Doyle

**Affiliations:** 1 Animal Welfare Science Centre, The University of Melbourne, North Melbourne, VIC 3051, Australia; phh@unimelb.edu.au (P.H.); rebecca.doyle@unimelb.edu.au (R.D.); 2 Faculty of Veterinary and Agricultural Science, The University of Melbourne, Werribee, VIC 3030, Australia; a.campbell@unimelb.edu.au; 3 Faculty of Veterinary and Agricultural Science, The University of Melbourne, Parkville, VIC 3052, Australia; srbarber@unimelb.edu.au

**Keywords:** animal-based indicators, animal welfare, on-farm welfare assessment, sheep

## Abstract

**Simple Summary:**

Sheep managed extensively can be exposed to several welfare challenges during the year, and the risk of some diseases can increase in warmer and wetter seasons. In this study, the welfare of Merino ewes was examined over a calendar year. The welfare of these animals, kept on a single farm with consistent management, varied substantially. Overall, the largest number of ewes experienced compromise and risk to welfare at weaning, indicating that this was the most vulnerable time. The main welfare issues identified were under and over feeding, ewe mortality, lameness, ecto-parasites (flystrike) and mastitis, all of which could be improved by modifying management practices, such as improved nutritional management and monitoring and better tail docking procedures. Future research must consider that significant variation in the on-farm welfare of ewes occurs during a calendar year, which needs to be accounted for when conducting on-farm assessments.

**Abstract:**

This study examined variation in the welfare of extensively managed ewes and potential welfare risks. A total of 100 Merino ewes (aged 2–4 years) were individually identified and examined at three key stages: pregnancy, lactation and weaning. Eight animal-based welfare measures were used to assess welfare: flight distance, body condition score (BCS), fleece condition, skin lesions, tail length, dag score, lameness and mastitis. Data were analysed by ANOVA and McNemar’s statistics. Overall, the average BCS of the group was in agreement with industry recommendations. However, a number of animals were classified with inadequate condition (either too thin or too fat) across the three observation periods. The presence of heavy dags was greatest at mid-lactation (87%, *P* < 0.0001), lameness was greatest at weaning (14%, *P* = 0.01), clinical mastitis was 1% annually, and five ewes were lost from the study. Ewes had better health at mid-pregnancy compared to mid-lactation and weaning. The main welfare issues identified were under and over feeding, ewe mortality, lameness, ecto-parasites (flystrike) and mastitis, all of which have the potential to be reduced with improved management practices. Future welfare assessment programs must consider that significant variation in on-farm welfare will occur in extensively managed systems and this needs to be accounted for when evaluating farms.

## 1. Introduction

The sheep industry is facing increasing financial and social pressures for assurances of good animal welfare and to maintain its ‘social license’ to operate [1]. In order to do this, further investigation of the current on-farm welfare status of sheep and potential welfare risks are needed [1,2]. Sheep that are farmed extensively in large flocks, in a year-round outdoor system and with low labour input, have the opportunity to live under more natural conditions and express natural behaviours [2]. This behavioural freedom creates opportunities for a better quality of life and a perception of positive welfare. Other aspects of extensive systems, however, do not necessarily guarantee good welfare, as extensively managed sheep can be at risk of several welfare challenges from a fluctuating environment and intermittent, unpredictable human interactions [3,4]. For example, fluctuation in environmental and climatic conditions produce important differences in food quality and availability. This, combined with changes in the sheep reproductive cycle, leads to variation in body condition over the course of the year [5,6]. Different seasons also increase the risk of some diseases in warmer and wetter periods, such as cutaneous myiasis [7] and foot rot [8]. In addition, the extensive nature of the industry makes feeding, predation control and prevention and treatment of diseases more difficult to monitor and promptly address, as there is limited individual care and supervision of animals [9]. Recent studies have suggested that the main on-farm welfare problems include poor nutrition (under and over-feeding), ecto- and endoparasites, reproductive health (e.g., mastitis, vulval cancer), lameness (and associated foot diseases) and mortality [2,4,10,11]. While the issues have been clearly identified, the extent of the issues are unknown as many sheep producers cannot accurately estimate the mortality rates in their farms [2]. 

Ewes’ health and welfare relates closely to both lamb and weaner survival, with healthy ewes in a good welfare state producing strong offspring at lower risk of chronic welfare compromise throughout their lives [12,13,14]. Ewes are vital to farm productivity, and maintaining ewes in good welfare has a significant impact on farm health and profitability. To date, most of the studies investigating sheep welfare have been conducted on lambs at transport [15,16], abattoirs [17,18], or in more intensive sheep farming conditions [1,6,19,20] and less attention has been given to the on-farm welfare of extensively managed ewes. This could be attributed to the general perception of positive welfare under extensive conditions but also to the complexity of assessing welfare in extensive systems, where time and labour limitations make it difficult to observe ewes in an undisturbed state, particularly in larger flocks and/or paddocks [7]. The present longitudinal study was designed to address this knowledge gap by examining variation in the on-farm welfare of one flock of extensively managed ewes and potential welfare risks over the course of three key stages; pregnancy, lactation and weaning.

## 2. Materials and Methods

### 2.1. Animals and Husbandry

This study is part of a longitudinal on-farm study that was performed in Victoria, Australia between July and December, 2015 [21]. The study was approved by the University of Melbourne ethics committee (ethical approval number 1513562.1). A total of 100 adult Merino ewes (aged 2–4 years), randomly selected from a larger flock of approximately 3000 breeding ewes, were individually identified by a unique ear tag number and examined at three-time points: mid-pregnancy (June), mid-lactation (October) and weaning (December). These reproductive periods were selected because they are known to be critical times for sheep welfare [1,7]. The ewes were managed under extensive commercial conditions, in a year-round outdoor system, and grazing a mix of annual and perennial pastures. The ewe sample size was selected based on a power calculation assuming 50% prevalence of the trait under observation (the proportion requiring the greatest sample size when observing binomial traits), a 95% confidence interval and precision of ±10%. This number was supported by the AWIN sheep protocol which recommends a sample of 92 animals when the farm size is ≥2000 breeding ewes [22]. Animals were not scanned for pregnancy before the study. However, farm records over the past three years were: 120% pregnancy rate, 100% lamb marking rate and 98% weaning rate. Upon physical examination of the udder at mid-lactation, it was found that 26% of the ewes (*n* = 25) failed to lamb or rear a lamb. This information was considered and the factor ‘reproductively active’ and ‘non-reproductively active’ was included in the analyses of the data.

### 2.2. Identification of Welfare Measures

The measures used to examine on-farm ewe welfare were derived from a review of the relevant scientific literature (including research papers, conference proceedings, theses and literature reviews published from 1994 to 2016). A total of 73 papers were identified, and those that included suitable animal-based measures for Australian extensive sheep farming conditions were evaluated for validity, reliability and practicality [23]. Valid welfare measures are those that provide meaningful information with respect to animal welfare, reliable measures provide repeatable outcomes when applied by different observers and practical measures are able to be collected in a large number of animals (≥100), in a short period of time, with minimum labour input. 

The welfare measures identified in the literature were compared to welfare measures identified in a large on-line survey of producers, industry experts and the general public [4], the AWIN sheep protocol [22], the Welfare Quality Assurance program [24], consultations with veterinarians and animal welfare scientists, and were then matched with the five domains of welfare compromise [25]. Table 1 shows the main animal-based welfare measures identified in the literature. The measures used in the present study were flight distance (FD), body condition score (BCS), fleece condition, skin lesions, tail length, dag score, lameness and mastitis. They were selected based on their reported validity (Table 1), and their reliability and feasibility reported in Munoz et al. [21]. 

### 2.3. Assessment of Seasonal Variation in the Welfare of Ewes

In order to perform the assessment, the ewes were managed in four groups of 25 animals. The assessment consisted of a group flight distance test (FD), and individual examination of BCS, fleece condition, skin lesions, tail length, dag score, lameness and mastitis. The assessment was performed using a holding pen (9 m × 8 m) and a single-file race within the farm’s regularly-used sheep yards, and was always conducted between 900 h and 1600 h.

The first step of the assessment was to measure flight distance (FD). The ewe’s response to an unfamiliar human was assessed with the assumption that the behavioral response of sheep to an individual will reflect the way in which they would respond to the farmer and/or external contractors and veterinarians, reflecting the process of stimulus generalization [26,27]. To test FD, a single human stimulus, always the same person (CM), quietly entered the pen holding a group of 25 ewes, walked around the perimeter and stood again at the entry point. From here, the observer waited for an ewe to be orientated towards her before approaching the animal. The ewe was approached by the observer in a standardised way (e.g., taking one step per second, maintaining the right arm in an angle of 45° in front of the body and the palm pointing towards the floor) [20,22]. The test ended when the ewe withdrew, defined as stepping away from the observer. Flight distance was estimated by counting the steps between the observer’s hand and the ewe’s head at the moment of withdrawal, and the behaviour of the ewe when approached by the observer was scored by using a 4-point score system as follows: a score of (0) if behaved calmly when approached; a score of (1) if there is some avoidance; a score of (2) if there is marked avoidance and struggling to escape; and a score of (3) if the ewe attempt to escape by jumping out of the pen [1]. Flight distance was measured in 5 ewes randomly selected from each of the four groups of 25 ewes, and was repeated at each stage. The ewes were not individually identified during the test, but the ewes tested were randomly selected from different locations within the group. The probability of not testing the same animal was 65%. 

The group of ewes was then moved to the single file race for individual examination of BCS, fleece condition, skin lesions, tail length, dag score, lameness and mastitis. The assessment criteria of the welfare measures are listed in Table 2. Mastitis was assessed by physical inspection of the udder and milk collection at mid-lactation and weaning. Ewes were restrained during this procedure in a purpose-built crate that allowed the ewe to remain in a standing position. At mid-lactation and weaning, all lactating ewes had approximately 35 mL of milk collected via hand milking. Prior to milk collection, teats were cleaned using an 80% ethanol solution. Milk was collected for both California Milk Test and somatic cell counts, with the person collecting the milk wearing gloves to eliminate any transfer of bacteria between human and sheep and vice versa.

### 2.4. Statistical Analysis

The welfare measures scoring scales consisted of numerical and categorical, ordinal data. Descriptive analysis of the data was performed in Excel^®^ before data were transferred to SAS statistical package (Statistical Analysis System, Release 9.4 2012; SAS Institute Inc., Cary, NC, USA). A two-way ANOVA (group composition × stage of reproduction) was used to examine differences in flight distance between the three reproductive stages examined. Multiple comparisons between means were performed using Fisher’s Least Significant Difference (LSD) test. Group composition refers to the four groups of 25 animals assessed each time. As the test depends on how the ewes behave towards the experimenter, ewes were not individually identified during the test. However, we identified the composition of each of the four groups at each stage, and this showed that different groups of sheep were formed at each time. In addition, a one-way ANOVA analysis was used to examine differences in BCS between ‘reproductively active’ and ‘non-reproductively active’ ewes.

A within animal comparison, to test differences in ewe welfare at the three stages, was performed by McNemar’s statistics (test for dependent dichotomous variables). McNemar’s statistic is used to examine the change in a binary repeated measurement, and can be used to test change in agreement over time [56]. To conduct this test, the seven animal-based welfare measures used for individual examination were transformed to binominal data. Body condition score was classified as adequate or inadequate according to industry recommended values [57], and ‘reproductively active’ and ‘non-reproductively active’ ewes were assessed independently. Fleece condition was transformed to adequate/inadequate (adequate fleece corresponding to ewes with a score of 0, see in Table 1). Skin lesions, dag score, lameness and mastitis were reclassified as present/absent. 

## 3. Results

### Assessment of Seasonal Variation in the Welfare of Ewes

The results of the individual welfare assessment are presented in Table 3. Five ewes were lost from the study period, with three ewes dying at lambing (reported dead by the farmer) and two presumed dead. Thus, different numbers of ewes were examined at mid-pregnancy (*n* = 100), mid-lactation (*n* = 96) and weaning (*n* = 95), and equals to a 5% mortality rate for ewes between mid-pregnancy and weaning. At mid-pregnancy, the mean BCS was 2.8 for ‘reproductively active’ ewes and 2.9 for ‘non-reproductively active’ ewes, and no statistical differences were found (*F* = 0.47, *P* = 0.49). At mid lactation, the mean BCS of the ‘reproductively active’ ewes was 3.1, while the mean BCS of the ‘non-reproductively active’ ewes was 3.3. The ANOVA analysis showed that the BCS between these groups were significantly different (*F* = 3.81, *P* = 0.05). At weaning, the mean BCS of the ‘reproductively active’ ewes was 2.9, whereas the mean BCS of the ‘non-reproductively active’ ewes was 3.1, and the ANOVA analysis showed that they were significantly different (*F* = 4.84, *P* = 0.03). In general, the number of animals classed with adequate/inadequate body condition varied over the course of the study. At mid-pregnancy, 29% of the ewes were below and 11% were above the recommended values. At mid-lactation, 10% of the ewes were below and 48% were above recommended values, and at weaning 12% of the ewes were below and 34% were above recommended values. A within animal comparison showed that the BCS of the ‘reproductively active’ ewes significantly decreased from mid-lactation to weaning (*P* = 0.03). The BCS of the ‘non-reproductively active’ ewes on the other hand, did not change over time (*P* = 0.73). Most of the ‘non-reproductively active’ ewes had a BCS of 3.5 ≥ (above recommended values) at mid-lactation and stayed in this category at weaning.

**Figure 1:**
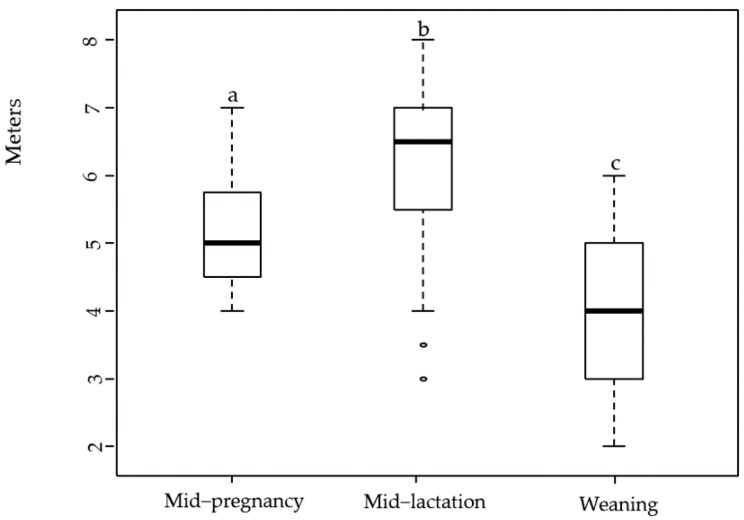
Median, minimum and maximum flight distance (FD) observed at mid-pregnancy, mid-lactation and weaning. Different letters indicate statistical difference (*P* < 0.05).

Fleece condition was overall adequate during the three periods examined. The percentage of ewes scored with adequate fleece condition ranged from 99% at mid-pregnancy to 95% at weaning. Skin lesions followed the same trend, with lesions mostly observed at weaning and related to cutaneous myiasis (flystrike; 86% of affected ewes, *n* = 6) and dermatophilosis (lumpy wool; 14% of affected ewes, *n* = 1). Dag scores were within the recommended values (scores 0–1) at mid-pregnancy and weaning, but significantly increased at mid-lactation (*P* < 0.0001) where 87% of the ewes had dag scores between 4 and 5. It was identified that 84 ewes that presented low dag score at mid-pregnancy (score 0–1) later presented high dag score at mid-lactation (score 4–5), and four ewes remained with a dag score of 3 at weaning, and their BCS ranged from 2.5 to 2.25. A total of 92% of the ewes at weaning (*n* = 87) had short-docked tails. Lameness increased significantly over time, from 5% (*n* = 5) at mid-pregnancy to 14% (*n* = 13) at weaning (*P* = 0.01). One ewe was lame at all three data collection times, and her lameness score increased from a score of 1 (clear shortening of stride) at mid-pregnancy to a score of 2 (not weight-bearing on affected limb) at weaning, and her BCS decreased from 2.75 at mid-pregnancy to 1.5 at weaning. At mid-lactation, no signs of clinical mastitis were observed, but the California Mastitis Test and somatic cell counts showed that 10% of the lactating ewes presented weak or distinct positive results to subclinical mastitis and somatic cell counts ranged from 29,000 to 4,022,000 cells/mL of milk. At weaning, the California Mastitis Test and somatic cell counts showed that 8% of the ewes presented weak or distinct positive results to subclinical mastitis, and somatic cell counts ranged from 38,000 to 17,177,000 cells/mL of milk. At this stage, 1% clinical mastitis was found and the affected ewe presented gangrenous mastitis, which corresponded to a score of 4. Ewes flight distance (FD) differed between each reproductive stage (*P* < 0.0001). The mean FD at mid-pregnancy was 5.15 m (SE ± 0.2), this significantly increased to 6.17 m (SE ± 0.3) at mid-lactation and decreased to 4.00 m (SE ± 0.2) at weaning (Figure 1). The behaviour score of the ewes when approached by the experimenter ranged from a score of 1 to a score of 2 during the study, and did not differ between reproductive stages. 

## 4. Discussion

The welfare of extensively farmed ewes on a single farm with consistent management varied substantially. This variation was seen both between individuals and within individuals during the study period. The animal welfare measures included in this study show both welfare compromise (evident at weaning) and welfare risk (high dag scores at mid-lactation). When assessing the biological functioning of the ewes, the largest number of ewes experienced compromise and risk to welfare at weaning. At this stage, 12% of the ewes were classed with a BCS below 2.5, 6% of the ewes presented skin lesions related to flystrike, there was 1% clinical mastitis and 14% lameness, indicating that this was the most vulnerable time. Results show that some welfare measures such as BCS, dag score and lameness can detect differences at key time points and predict the likelihood of individual ewes experiencing future welfare compromise. While identified welfare issues may be exacerbated by the season, there is potential for improvement with changed management practices at each stage of the reproductive cycle. As a result, we discuss the results of the welfare assessment in conjunction with management activities that could reduce these issues. 

In this study, the average BCS of ewes studied was in agreement with industry recommendations. However, a number of animals were classified with inadequate condition, either too thin or too fat, across the three observation periods. Body condition scoring of sheep has been validated [7,21,28,58] and is widely accepted as an animal-based measure that can be used to monitor past nutritional management and potential welfare risks of sheep [7,59]. It is possible that some of the pregnant ewes classed with low body condition at mid-pregnancy, particularly the ones classed with a BCS < 2.5, may have experienced reduced placental development and foetal growth [60,61], and were more susceptible to metabolic diseases, such as pregnancy toxemia [59]. The ewes scored fat at mid-pregnancy, besides being at more risk of metabolic diseases, may also be more likely to experience difficulties at lambing [62]. Results from udder inspection at mid-lactation detected that 26% of the flock failed to lamb or rear lambs, suggesting that either a significant number of ewes were not pregnant, had lost pregnancies or lost lambs at or soon after birth. Based on the farm history on reproductive rates and results for BCS in the study, it is likely that a large number of ewes had lost lambs soon after birth [63]. By tracking the same ewes over the study, a 5% mortality rate between mid-pregnancy and weaning was identified. Generalizing this figure across the whole flock, it would translate to 150 ewe deaths in the 6 months of the study, which has welfare and economic consequences. Previous studies suggests that lack of information on farm mortality could produce a disconnection between perceived and actual ewe mortality, and this underestimates welfare issues on the farm and may lead to a lack of interest by producers to address particular welfare concerns [10]. To comprehensively assess ewe welfare, measures such as BCS cannot be taken in isolation, and they need to be integrated with further details of farm records, management and resources. The fact that a consistent number of animals had inadequate body condition during the study, making them susceptible to several diseases, highlights the importance of appropriate nutritional management to reduce individual welfare compromise. Overall, the main welfare risks identified in this study may have arisen because of both under and over feeding. Condition scoring at key times and pregnancy scanning of ewes to identify ewes that are not pregnant, or carrying single or multiple lambs allows farmers to use a targeted feeding approach to ensure that ewes carrying multiple lambs receive adequate levels of nutrition [63]. 

The presence of heavy dags is an important indicator of potential welfare risk of flystrike. A ewe with a dag score of 4 is seven times more susceptible to flystrike than a ewe with a dag score of 1 [64]. In addition, the presence of heavy dags could also indicate current welfare compromise as scouring and dags could be related to worm burdens or larval challenge in ewes with depressed immunity, but it could also be reflection of rapid changes in the diet, which causes nutritional scours. In the present study, a large percentage of ewes (87%) were classed with high dag scores (scores 4 to 5) at mid-lactation. A possible cause for this increase may be related to a rapid change to a high-quality pasture with high water content of spring grass, as the number of ewes presenting high dag scores significantly decreased by weaning (late spring). This decrease in scouring and dags may be indicating an adaptation to the spring grass, however, four ewes remained with a dag score of 3 until weaning, and they had a BCS of ≤ 2.5 which may be an indication of worm burdens or larval challenge or other gastrointestinal condition [7,35,64]. Parasitised sheep usually present poor body condition, low growth rates, reduced wool and milk production, and are more susceptible to other diseases such as flystrike [59,65]. In addition, the fact that 6% of the group presented skin lesions related to flystrike highlights the importance of early treatment in extensive systems. Effective management of dags and scouring is essential. Dags can be managed by ‘crutching’ (wool from around the tail and between the rear is shorn), but it is important to perform worm egg counts to monitor worm burdens and improve worm management and drench programs to understand the cause and appropriate treatment for any scouring [7,64,66].

Lameness is a worldwide problem in sheep, and it is an important health and welfare concern due to the pain, discomfort and debilitation that it causes [36]. In this study, the percentage of lame ewes increased significantly from 5% at mid-pregnancy to 14% at weaning, similar to a previous study investigating seasonal variation in lameness in extensive systems in the UK [8]. An increase in lameness at weaning may be associated with footrot lesions [41], hoof overgrowth, and foot abscess due to environmental conditions such as warmer and wetter conditions [67,68]. In some areas of South Eastern Australia, footrot is considered an endemic disease, with more than 20% of the farms affected [69]. In this study, it was not possible to identify the cause of lameness on the farm since the ewes were not individually restrained for further inspection. Although this assessment cannot give details of what is causing lameness, a high lameness percentage can alert farmers to a musculoskeletal problem affecting animal welfare that warrants more investigation. Previous studies have shown that lame sheep may experience pain and discomfort which reduces mobility, feed intake and weight gain, decreasing body condition and wool production [37,70]. The fact that a number of ewes were lame at weaning (14%) emphasize the importance of early intervention and frequent monitoring to reduce individual animal welfare compromise [37].

Overall, 92% of the ewes assessed had short-docked tails (less than three palpable joints) when compared with recommended guidelines [54]. Since the vast majority of the ewes had short docked tails in this study, relationships between tail length and welfare issues were not examined. However, previous studies have demonstrated that sheep with short-docked tails may experience more pain and poor healing after tail docking and may be more susceptible to bacterial arthritis [71], rectal prolapse, dags, flystrike [72] and vulva cancer [54]. By using tail length as an indicator of past welfare compromise and future welfare risks, it is possible that a number of the ewes in the flock experienced or would be at risk of one or more of these welfare compromises.

Mastitis is increasingly recognised as an important health and production concern in extensively-managed sheep flocks. Mastitis can be categorised as clinical or sub-clinical and the majority of the cases occur in the month post lambing or before weaning, and/or following adverse weather conditions [43]. In the present study, no signs of clinical mastitis were observed at mid-lactation, but 10% of lactating ewes had subclinical mastitis. At weaning, 8% of the ewes presented subclinical mastitis and 1% clinical mastitis (gangrenous mastitis). These results are in agreement with previous studies that have reported 7% subclinical mastitis and 1–2% clinical mastitis in Merino ewes [73]. However, since little is known about the prevalence and incidence of mastitis in Merino flocks, it is possible that figures may be higher than currently identified. It has been shown that ewes affected may experience pain and discomfort, which can result in body condition loss, lameness and mismothering of lambs [55]. While it represents compromised welfare and productivity of ewes and their lambs, assessing mastitis on-farm in sheep that are not managed as milking flocks, is time-consuming, labour-intensive and frequently impractical. Since this study was conducted in one flock, further research is needed to describe mastitis prevalence in extensively-managed wool and meat flocks, and its impact on ewe and lamb welfare.

Flight distance (FD) was used to examine the quality of the human-animal relationships (HAR) of extensively managed ewes [20,74]. There is currently limited information on these relationships in animals farmed extensively, where human-animal interactions are infrequent and often stressful for both animals and farm staff e.g., at castration, tail docking and shearing [5,74]. The ewes examined in the present study had a mean FD of 5.15 m at mid-pregnancy, 6.17 m at mid-lactation and 4 m at weaning. In addition, ewes usually presented marked avoidance to the experimenter, indicating fear towards humans, particularly at mid-lactation. The FDs and the behaviour scores recorded in this study were higher than what has been observed in intensively managed ewes [1,20] but in agreement with results obtained by Hutson [75] who examined FD in sheep farmed extensively. It is possible that results observed in the present study do not necessarily indicate a negative HAR, as the likelihood of having a short FD, in systems where animals do not frequently receive human contact, would be low, suggesting that the current interpretation of this measure is unrealistic for extensive systems. While only one flock at one farm was assessed, the variation observed in FD suggests that this measure may be influenced by the time-point of the assessment, and/or ewes’ familiarity with the test/experimenter. Previous studies have shown that FD varies significantly between pregnant and non-pregnant ewes [76,77]. Ewes with lambs at foot tend to be more reactive to external stimulus to protect their off spring [78], which could explain the significant increase in FD at mid-lactation. Following the removal of lambs, FD reduced significantly at weaning to a level lower than mid-pregnancy. This may be related to habituation to the testing situation as well as the experimenter of this study, as it has been shown that repetitive exposure or habituation to certain procedures changes the animal’s response/perception of that stimulus [79]. The test performed in this study is consistent with other human-approach tests for livestock; however, results in this study indicate that the short FD suggested for intensively managed sheep cannot be extrapolated to sheep managed extensively and that the time-point of the assessment may affect FD. A limitation of this group test is that ewes were not individually identified during the test, and therefore, some ewes may have been assessed repeatedly during the study. Further work is needed to validate a reliable and practical on-farm assessment of fear of humans that could be applied to extensive systems.

## 5. Conclusions

When assessing the biological functioning of the ewes, the largest number of ewes experienced compromise and risk to welfare at weaning than at mid-pregnancy and mid-lactation which needs to be considered when deciding key times to conduct on-farm welfare assessments. The main welfare issues identified were poor nutrition (under and over feeding), ewe and potentially lamb mortality, lameness, flystrike and mastitis. The main welfare risks identified were the possibilities of metabolic diseases and dystocia due to inadequate BCS, flystrike from heavy dags, and reproductive problems due to short tail length. The majority of the welfare issues and potential welfare risks identified in this study could be improved by modifying management practices, such as improved nutritional management and monitoring and better tail docking procedures. Further information on disease records, early detection and intervention of sick animals and mortality records need to be incorporated in the assessment to examine prospective welfare issues. Further research must consider that significant variation occurs at key periods of the sheep production cycle which needs to be accounted for when conducting on-farm welfare assessments.

## Figures and Tables

**Table 1:** Animal-based welfare measures identified for extensively managed ewes.

Five Domains Principles	Category	Indicator	Animals	Validity	Reference
**Nutrition**	Nutrition	Body condition score	Lambs/ewes	H	[1,7,28,29,30]
	Feed and water	Rumen fill	Lambs/ewes	L/M	[31,32]
**Environmental challenge**	Shade and shelter	Panting	Ewes	H	[19,33,34]
		Fleece cleanliness	Ewes	H	[1,7,19,20]
**Disease, injury, functional impairment**	Gastrointestinal health	Faecal soiling	Ewes	H	[6,7,35]
	Integument alterations	Fleece condition	Ewes	H	[1,6,7]
		Skin lesions	Ewes	H	[1,7,20]
	Foot condition and lameness	Foot-wall integrity	Ewes	L/M	[7,36,37,38,39]
		Hoof overgrowth	Ewes	M	[7,20,37,40]
		Gait score	Ewes	M/H	[6,7,19,20,41,42]
	Reproductive health	Mastitis	Ewes	M/H	[7,43]
		Tail length	Lambs/ewes	H	[7,44]
	Systemic disease	Social withdrawal	Ewes	M	[6]
**Behavioural restriction**	Agonistic behaviour	Aggression	Ewes	M	[5,45,46]
	Abnormal behaviour	Stereotypies	Ewes	H	[46,47]
**Mental state**	Behaviour	QBA *	Wethers/ewes	M/H	[6,31,48,49,50,51]
	HAR ^#^	Flight distance	Lambs/ewes	L/M	[1,20,52,53]

* QBA refers to Qualitative Behaviour Assessment; ^#^ HAR refers to human-animal relationships. Validity scale was as follows: H = high, M = moderate and L = low. High validity was given to animal-based measures validated in previous research, medium validity was given to measures without a reliable method of assessment and low validity was given to measures that have been suggested in scientific literature but without evidence that they assess welfare.

**Table 2:** Animal-based welfare measures used to assess the welfare of extensively managed ewes.

Welfare Measure	Assessment Criteria
Flight distance	Flight distance was estimated by counting the steps between the observers’ hand and the ewes’ head at the moment of withdrawal [20,22]. The behaviour of the ewe when approached by the observer was scored by using a 4-point score system as follow: (0) behaved calmly when approached; (1) some avoidance; (2) marked avoidance and struggling to escape; and (3) attempts to escape by jumping out of the pen [1].
Body condition score	Scored on a 5 point scale from 1 (thin) to 5 (obese), using a quarter-unit precision. Sheep were assessed by palpation of the backbone, muscle and short ribs [28,30]
Fleece condition	Scored on a 3 point scale: (0) good fleece condition, when parted, the fleece has no lumpiness or signs of ectoparasites; (1) some fleece loss, small shed or bald patches of no more than 10 cm diameter. When parted, the fleece may have some lumpiness or scurf, little evidence of ectoparasites; and (2) significant fleece loss with bald patches of greater than 10 cm in diameter, clear evidence of ectoparasites [22].
Skin lesions	Assessed by recording number, location, type and size of the skin lesions. Lesions were classified as cuts, open wounds, old wounds or scars and abscesses.
Tail length	Scored on a 2 point scale: (0) the tip of the vulva is covered by the tail when held down; (1) the tail is over-shortened or almost not present, or if the vulva and anus cannot be covered [22,54]
Dag score	Scored on a 6 point scale: (0) no evidence of faecal soiling; (1) very light soiling on the breech area; (2) Moderate dag on the breech area extending ventrally; (3) Severe dag predominantly on the breech area, extending ventrally and dorsally over the tail some soiling and dag around anus; (4) excessive dag on the breech area and on the hind legs; (5) Very severe dag on the breech area and on the hind legs or below the level of the hocks [35]
Lameness	Scored on a 4 point scale: (0) not lame; (1) clear shortening of stride with obvious head nodding or flicking as the affected limb touches the floor; (2) clear shortening of stride with obvious head nodding and not weight-bearing on affected limb whilst moving; (3) reluctant to stand or move [22].
Mastitis	Scored on a 5 point scale: (0) normal udder; (1) a small fibrotic lesion within the mammary tissue, normal secretion; (2) A more extensive fibrosis of the udder. Milk ranged from normal to purulent; (3) Extensive swelling of the udder, that could be abscessed or ruptured; (4) Peracure mastitis. Complete udder involvement with severe inflammation. Secretion from serum-like to purulent. Mammary lymph nodes enlarged. Body temperature elevated [55].

**Table 3:** Mean, minimum and maximum scores, recommended values (RV), percentage of ewes identified within recommended values (% within RV) and above recommended values (% above RV) at mid-pregnancy, mid-lactation and weaning; standard deviation in parentheses.

Measures	Mean Score	Min	Max	RV	% within RV	% above RV
Mid-pregnancy						
BCS	2.83 (0.45) *	2	3.75	2.7–3.3 ^#^	60%	11%
Fleece condition	0.02 (0.14)	0	1	0 ^a^	98%	2%
Skin lesions	0.00 (0.14)	0	1	0 ^a^	99%	1%
Dag score	0.54 (0.76)	0	3	0–1 ^a^	99%	1%
Lameness	0.06 (0.27)	0	2	0 ^a^	95%	5%
Mid-lactation						
BCS	3.11 (0.42) *	2.5	4	2.7–3 ^#^	42%	48%
Fleece condition	0.03 (0.17)	0	1	0 ^a^	97%	3%
Skin lesions	0.01 (0.08)	0	1	0 ^a^	92%	8%
Dag score	3.94 (0.89)	1	5	0–1 ^a^	13%	87%
Lameness	0.06 (0.28)	0	2	0 ^a^	95%	5%
Clinical mastitis	0.00 (0.00)	0	0	0 ^a^	100%	0%
Weaning						
BCS	2.88 (0.52) *	1.5	3.75	2.5–3 ^#^	54%	34%
Fleece condition	0.09 (0.39)	0	2	0 ^a^	95%	5%
Skin lesions	0.07 (0.27)	0	1	0 ^a^	93%	7%
Dag score	0.94 (0.25)	0	3	0–1 ^a^	98%	2%
Lameness	0.13 (0.35)	0	1	0 ^a^	86%	14%
Clinical mastitis	0.06 (0.49)	0	4	0 ^a^	99%	1%

* Mean BCS of ‘reproductively active’ ewes; ^#^ Industry recommendations of BCS [57]; ^a^ Score recommended by [22].
